# Multiple obesity indices suggest a close relationship between obesity and constipation: evidence from NHANES

**DOI:** 10.1186/s12889-024-18647-y

**Published:** 2024-05-09

**Authors:** Nengjun Xiang, Lulu Xu, Haihua Qian, Dan Zhang

**Affiliations:** 1 Department of Anorectal Surgery, The Affiliated Hospital of Nanjing University of Chinese Medicine, Nanjing, China; 2 Department of General Surgery, The Affiliated Hospital of Nanjing University of Chinese Medicine, Nanjing, China

**Keywords:** Constipation, Obesity, NHANES, National Health and Nutrition Examination Survey

## Abstract

**Objective:**

This study aims to investigate the relationship between obesity and constipation among American adults.

**Methods:**

Our study leveraged data from the National Health and Nutrition Examination Survey (NHANES). This comprehensive approach enabled us to summarize the weighted prevalence rates of obesity in adults. To further deepen our understanding, we employed a variety of analytical methods. These included multivariable logistic regression, subgroup analysis and restricted cubic splines. Through these methodologies, we were able to effectively evaluate the correlation between various obesity indicators and constipation, offering new insights into this complex relationship.

**Results:**

The weighted prevalence of constipation stands at 9.42%. Notably, an increased risk of constipation is linked with a BMI (body mass index) exceeding 28 kg/m2, WSR (waist-stature ratio) that is either between 58.3 and 64.8 or above 64.8, as well as a LAP (lipid accumulation products) ranging from 50.8 to 90.1. In contrast, a reduced risk of constipation is associated with WWI (weight-adjusted-waist index) that falls between 0.015 and 0.020, exceeds 0.020, and without the presence of central obesity (*P* < 0.05). Restricted cubic spline analysis, a significant non-linear relationship was discerned between BMI, WSR, and LAP in relation to constipation.

**Conclusions:**

This pioneering large-scale study explores the relationship between various obesity indices and constipation. It reveals that reducing the BMI, WSR, LAP and waist circumference can decrease the risk of constipation. Conversely, a higher value of WWI correlates with a lower constipation risk, and this remains true even after adjusting for a wide range of variables.

## Introduction

Global obesity rates are on the rise [1], presenting a significant challenge to public health. According to the ‘2017 Global Nutrition Report’ more than 2 billion adults worldwide are either overweight or obese. This concerning trend is rooted in the fact that obesity isn’t just linked to an increased risk of cardiovascular diseases, diabetes, and specific cancers; it also serves as a crucial predictor of metabolic disorders. Furthermore, obesity shows a significant association with various digestive system ailments, primarily attributable to the excessive accumulation of fat [2].

Among these conditions, chronic constipation stands out as a common gastrointestinal disorder, affecting roughly 16% of adults worldwide [3]. It deserves attention due to its adverse impact on quality of life and its potential to lead to severe health complications. Research, including a study conducted on Italian residents, has suggested a higher prevalence of constipation among obese individuals [4], potentially linked to their dietary habits and levels of physical activity [5]. Obesity may indirectly contribute to constipation through mechanisms such as the alteration of intestinal hormone secretion, impaired intestinal motility, and inflammatory responses. However, other studies, particularly those involving American adults, propose a contrasting view that there is no direct correlation between being overweight and experiencing constipation [6, 7]. This discrepancy underscores the need for further research in this field.

Visceral obesity is typically quantified using computed tomography (CT) or magnetic resonance imaging (MRI) [8]. To avoid high costs and exposure to electromagnetic radiation, body mass index (BMI) and waist circumference (WC) are more commonly employed to assess visceral obesity [9, 10]. However, recent years have witnessed the emergence of indices that combine anthropometric measurements with lipid values, such as the visceral adiposity index (VAI) and lipid accumulation products (LAP). These indices have demonstrated higher accuracy in identifying visceral obesity [11].

The primary aim of our study is to investigate the relationship between various definitions of obesity, including VAI, weight-adjusted waist index (WWI), waist-stature ratio (WSR), LAP, BMI, and waist-based central obesity, in relation to constipation while controlling for confounding factors. LAP, a cost-effective index, is frequently used to assess obesity from multiple perspectives [12]. Our multidimensional approach to obesity assessment encompasses various aspects, ranging from fat distribution to metabolic risk, offering a comprehensive perspective on how obesity impacts intestinal health.

This paper systematically reviews and analyzes the correlation between these obesity indicators and the prevalence of constipation. Our objective is to elucidate the individual and combined effects of these indicators on the development of constipation. To accomplish this, we leverage a large dataset from the National Health and Nutrition Examination Survey (NHANES) database, along with genome-wide association study (GWAS) pooled data, to evaluate the association between constipation and obesity in American adults aged 20 and above. Through the comparison of various obesity indicators, we aim to pinpoint which ones are most closely associated with the risk of constipation, thus providing valuable insights for clinical prevention and treatment strategies, guiding clinical practice, and informing future research directions.

## Methods

### Data source and participants

**Fig. 1:**
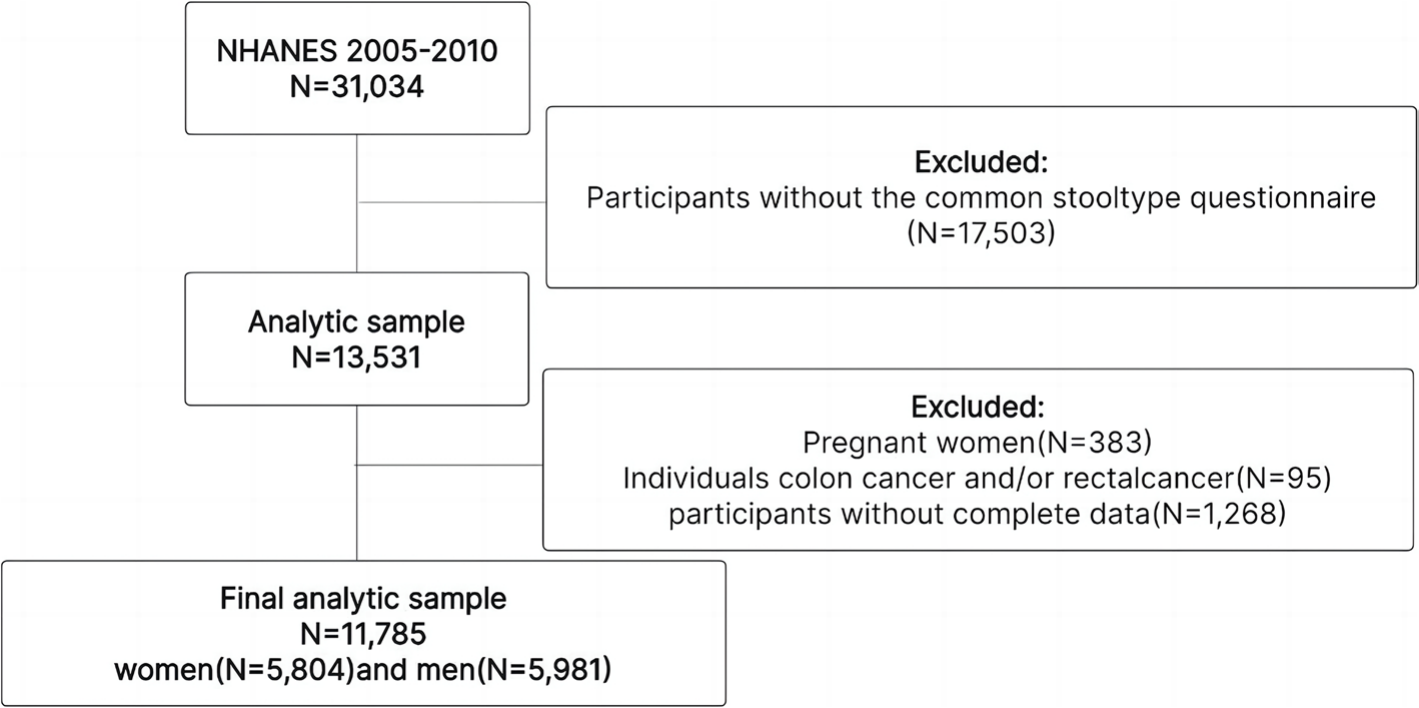
Flowchart of the systematic selection process

The National Health and Nutrition Examination Survey (NHANES), conducted by the National Center for Health Statistics under the Centers for Disease Control and Prevention (Atlanta, Georgia, USA), is a cross-sectional study focusing on a nationally representative sample of the non-institutionalized US population. Employing a complex, stratified, multistage, probability cluster design, it selects non-institutionalized individuals from the US. All participants gave written informed consent, as mandated by the Ethics Review Committee of the National Center for Health Statistics. Our current analysis zeroes in on participants aged ≥ 20 years from three NHANES cycles, all of whom completed the intestinal health and dietary assessment questionnaire. From 2005–2010, out of the 31,034 NHANES participants, 13,531 filled out the dedicated questionnaire on intestinal health. After excluding pregnant individuals (*n* = 383), those diagnosed with colorectal cancer (*n* = 95), and those with missing data (*n* = 1,268), the study considered a final sample of 11,785 participants, split between 5,981 males and 5,804 females. The flow chart of the systematic selection process is shown in Fig. 1.

### Measures

#### Adiposity measures

At the Mobile Examination Center, trained health technicians employ standardized methods detailed in a procedural manual to measure weight, height, and waist circumference. Each measurement session involves a health technician and an accompanying recorder. Health technicians also draw blood samples from the antecubital vein of all study participants. Examination indicators encompass high-density lipoprotein cholesterol (HDL) and fasting triglycerides (TG). To investigate and compare the correlations between various obesity indices and the presence of constipation, we utilized data from the NHANES database and selected six distinct anthropometric indices as obesity indicators: BMI, VAI, WWI, WSR, LAP, and WC. These six indices were chosen for their ease of measurement and widespread recognition [13–15].

BMI is calculated using the following formula: BMI = weight (kg) / (height (m))^2. Based on BMI values, the World Health Organization classifies individuals as underweight (BMI < 18.5), normal weight (BMI 18.5–24.9), overweight (BMI 25–29.9), or obese (BMI ≥ 30). VAI is an integrated index that considers BMI, WC, TG, and HDL: for males, VAI = [WC (cm) / 39.68 + (1.88 * BMI)] * (TG (mmol/L) / 1.03) * (1.31 / HDL (mmol/L)); for females, VAI = [WC (cm) / 36.58 + (1.89 * BMI)] * (TG (mmol/L) / 0.81) * (1.52 / HDL (mmol/L)) [16]. WWI is computed as WC in centimeters divided by the square root of weight in kilograms. WSR represents the ratio of waist circumference to height [17]. LAP serves as an indicator to assess lipid accumulation, incorporating WC and triglycerides (TG): for males, LAP = (WC (cm) - 65) * TG (mmol/L); for females, LAP = (WC (cm) - 58) * TG (mmol/L) [18]. WC, VAI, WWI, and WSR each employ the 25th, 50th, and 75th percentiles (P25, P50, and P75) as threshold values. Central obesity is defined as a waist circumference (WC) ≥ 88 cm in females or ≥ 102 cm in males.

#### Constipation

NHANES conducted an intestinal health questionnaire from 2005 to 2010 during which data regarding stool frequency and consistency were recorded. This data played a pivotal role in defining constipation within the scope of this study. Notably, both of these measures were considered in the establishment of constipation criteria.

The primary definition of constipation was derived from participants’ self-reports concerning their usual stool characteristics, categorized as either Type 1 (hard, nut-like lumps) or Type 2 (lumpy, sausage-like stools) on the Bristol Stool Form Scale.

Additionally, a secondary definition of constipation was formulated based on participants’ responses to a specific question regarding stool frequency: “How often do you typically have a bowel movement per week?” Individuals falling under the constipated category were those who reported having bowel movements less than or equal to two times per week. Conversely, individuals with bowel movements occurring more than twice a week were classified as non-constipated.

#### Covariates

Several variables were evaluated as potential covariates due to their hypothesized or previously demonstrated association with constipation and obesity. These variables included the intake of energy [8], fiber [9], fat [10], phosphorus [11], magnesium [12] and selenium [13], as well as factors like depression scores [14], sleep duration [15], and exercise levels [16]. In our sociodemographic assessment, we considered age (treated as a categorical variable), gender, race/ethnicity (spanning categories from Mexican–American to non-Hispanic Black), educational attainment, and PIR (with two distinct ranges).

For behavioral risk factors, we examined smoking and drinking habits, physical activity intensity, and sleep patterns. We categorized individuals into never smokers and regular smokers based on their lifetime cigarette consumption. Those consuming a minimum of 12 alcoholic drinks annually were labeled as drinkers. Physical activities were bifurcated into intense (e.g., heavy lifting or construction work causing significant breath or heart rate increase) and non-intense types. Sleep duration was segmented into four categories, ranging from very short to extended sleep durations.

Medical conditions were also considered. Diabetes was identified based on a physician’s diagnosis, while hypertension was identified through professional medical advice. Depression status was ascertained using the NHANES mental health questionnaire, specifically relying on the Patient Health Questionnaire 9 scale, a validated tool for depression assessment [17].

### Statistical analyses

We conducted data analysis using R (version 3.4.3, 2021–12-21, R Foundation; http://www.r-project.org) and Stata (MP 17.0). Considering sampling weights, we estimated sampling errors through Taylor series linearization. Using NHANES census data from 2005–2010, we summarized the weighted prevalence of US adults in proportional units. We examined the baseline characteristics of study individuals through descriptive analysis, stratifying by the presence of constipation. Continuous variables were presented as weighted means ± standard errors (SE), while categorical variables were represented as counts (weighted percentages, %). Chi-square statistics were used for categorical variables, and analysis of variance for continuous variables.

To assess the associations between various obesity indices and the prevalence of constipation, we employed a survey multivariable logistic regression to calculate adjusted odds ratios (AORs) and 95% confidence intervals (CIs). Our analysis consisted of three models. The crude model did not adjust for categorical variables. Model 2 adjusted for age, gender, and ethnicity. Model 3, based on Model 2, further adjusted for gender, age, ethnicity, education, ratio, diabetes, smoking, alcohol use, hypertension, sleep duration, depression, physical activity, and dietary intake of energy, protein, carbohydrates, dietary fiber, total fat, total saturated fatty acids, total monounsaturated fatty acids, and total polyunsaturated fatty acids. *P* < 0.05 was considered statistically significant.

To study the relationship between six obesity indices and constipation, we employed a restricted cubic spline regression model with three knots located at the 25th, 50th, and 75th percentiles. Our analysis further considered the relationship between constipation and various obesity indices, stratified by gender, age, ethnicity, education, PIR, diabetes, smoking status, alcohol consumption, hypertension, sleep duration, depression, and vigorous physical activity.

## Results

### Demographic characteristics

The demographic characteristics, as shown in Table 1, encompassed a total of 11,785 participants (5,981 males and 5,804 females) aged 20 and above who were surveyed over six NHANES cycles. The unweighted and weighted prevalence rates for the total participants in the sample were 9.83% and 9.12%

**Table 1:** Baseline characteristics of the study population from national health and nutrition examination survey 2005–2010 (using the stool consistency definition of constipation), weighted

**Characteristics**	**Overall [n(%)]**	**Constipation [n(%)]**	**No constipation [n(%)]**	***P*-value**^**b**^
Total participants	11,785 (100)	9.1	90.9	**< 0.001**
Sex				**< 0.001**
Male	5981 (49.6)	25.6	52.0	
Female	5804 (50.4)	74.4	48.0	
Age (years)	46.00 ± 0.34	43.00 ± 0.55	46.00 ± 0.36	**< 0.001**
20–29	1939 (18.3)	25.3	17.6	**< 0.001**
30–39	1986 (18.4)	17.1	18.5	
40–49	2162 (21.5)	20.3	21.6	
50–59	1878 (19.3)	17.8	19.5	
60–69	1927 (12.2)	9.6	12.5	
70–79	1255 (7.0)	5.9	7.1	
> 80	638 (3.3)	3.9	3.2	
Ethnicity				**< 0.001**
Mexican American	2091 (7.8)	8.1	7.7	
Other Hispanic	936 (4.1)	4.8	4.0	
Non-Hispanic White	6038 (72.9)	67.0	73.5	
Non-Hispanic Black	2259 (10.2)	15.9	9.6	
Other Race - Including Multi-Racial	461 (5.1)	4.2	5.2	
Education				**< 0.001**
Below high school	3136 (17.3)	21.2	16.9	
High school	2820 (24.1)	29.6	23.5	
Above high school	5829 (58.6)	49.2	59.6	
PIR	3.23 ± 0.04	2.34 ± 0.07	3.33 ± 0.04	**< 0.001**
< 2	5297 (31.2)	42.4	30.1	**< 0.001**
≥ 2	6488 (68.8)	57.6	69.9	
Diabetes				0.051
Yes	1270 (7.6)	7.9	7.5	
No	10,300 (90.8)	91.5	90.7	
Borderline	215 (1.7)	0.7	1.8	
Smoking				**< 0.001**
Yes	5675 (47.6)	42.2	48.1	
No	6110 (52.4)	57.8	51.9	
Alcohol use				**< 0.001**
Yes	8586 (77.0)	66.9	78.0	
No	3199 (23.0)	33.1	22.0	
Hypertension				**0.015**
Yes	4040 (30.0)	26.7	30.3	
No	7745 (70.0)	73.3	69.7	
Sleep duration				**< 0.001**
< 5 h/night	685 (4.9)	6.4	4.7	
5–6 h/night	3931 (31.3)	36.1	30.8	
Normal	6343 (57.4)	49.2	58.2	
≥ 9 h/night	826 (6.5)	8.3	6.3	
Depression				**< 0.001**
No	10,600 (91.7)	84.5	92.5	
Yes	1185 (8.3)	15.5	7.5	
Physical activity				**0.025**
Vigorous physical activity	2831 (27.0)	22.8	27.4	
No vigorous activity	8954 (73.0)	77.2	72.6	
Daily dietary intake				
Energy (kcal/d)	2027 (1511,2722)	1796 (1361,2440)	2047 (1527,2758)	**< 0.001**
Protein (gm/d)	77.3 (55.2,106.1)	65.4 (47.0,91.3)	78.4 (56.2,107.6)	**< 0.001**
Carbohydrate (gm/d)	240.4 (176.0,326.3)	227.8 (168.4,307.3)	241.6 (176.9,328.5)	**< 0.001**
Dietary fiber (gm/d)	14.4 (9.7,20.8)	12.4 (7.8,17.9)	14.7 (9.9,21.1)	**< 0.001**
Total fat (gm/d)	75.6 (51.0,107.4)	66.5 (44.0,93.1)	76.8 (52.1,108.6)	**< 0.001**
Total saturated fatty acids (gm/d)	24.3 (15.7,36.0)	21.8 (13.9,31.7)	24.7 (16.0,36.4)	**< 0.001**
Total monounsaturated fatty acids (gm/d)	27.3 (18.2,39.6)	23.4 (15.4,34.6)	27.8 (18.3,40.1)	**< 0.001**
Total polyunsaturated fatty acids (gm/d)	15.7 (10.0,23.3)	13.4 (8.0,19.9)	15.9 (10.2,23.5)	**< 0.001**
Cholesterol (mg/d)	227 (134,384)	194 (111,332)	231 (137,390)	**< 0.001**
Phosphorus (mg/d)	1276 (927,1744)	1101 (787,1525)	1297 (944,1764)	**< 0.001**
Magnesium (mg/d)	279 (204,375)	236 (168,323)	283 (208,382)	**< 0.001**
Selenium (mcg/d)	103 (70,143)	88 (60,119)	105 (71,146)	**< 0.001**
Caffeine (mg/d)	135 (36,281)	92 (15,240)	140 (39,284)	0.186

Continuous variables are presented as mean ± SE or median (interquartile range), and categorical variables are presented as n (%)

*SE* Standard error

^b^*P*-value based on survey t-test for binominal groups, and based on chi-square test in the qualitative variables. *P*-values in bold indicate statistical significance

The mean age of all participants was 46.00 ± 0.34 years. The majority fell into the 40–49 age group, comprising 2,162 individuals (21.5%), followed by the 30–39 age group with 1,986 individuals (18.4%). The distribution of participants was relatively even across age groups.

Regarding ethnic backgrounds, most of the participants were non-Hispanic white, totaling 6,038 individuals (72.9%). This was followed by 2,259 individuals (10.2%) of Non-Hispanic Black descent, 2,091 individuals (7.8%) of Mexican American descent, and 1,397 other individuals (9.2%).

Participants defined as constipated exhibited certain characteristics. They were more likely to be female (74.4%), non-Hispanic white (67.0%), have higher education levels (49.2%), lower poverty income ratio (57.6%), non-diabetic (91.5%), non-smoker (57.8%), be drinkers (66.9%), non-hypertensive (73.3%), have moderate sleep duration (49.2%), non-depressed (84.5%), non-vigorous exercisers (77.2%), and had lower daily intake of energy, protein, carbohydrate, dietary fiber, total fat, total saturated fatty acids, total monounsaturated fatty acids, total polyunsaturated fatty acids, cholesterol, phosphorus, magnesium, and selenium (*P* < 0.05).

### Relationship between obesity and constipation

**Fig. 2:**
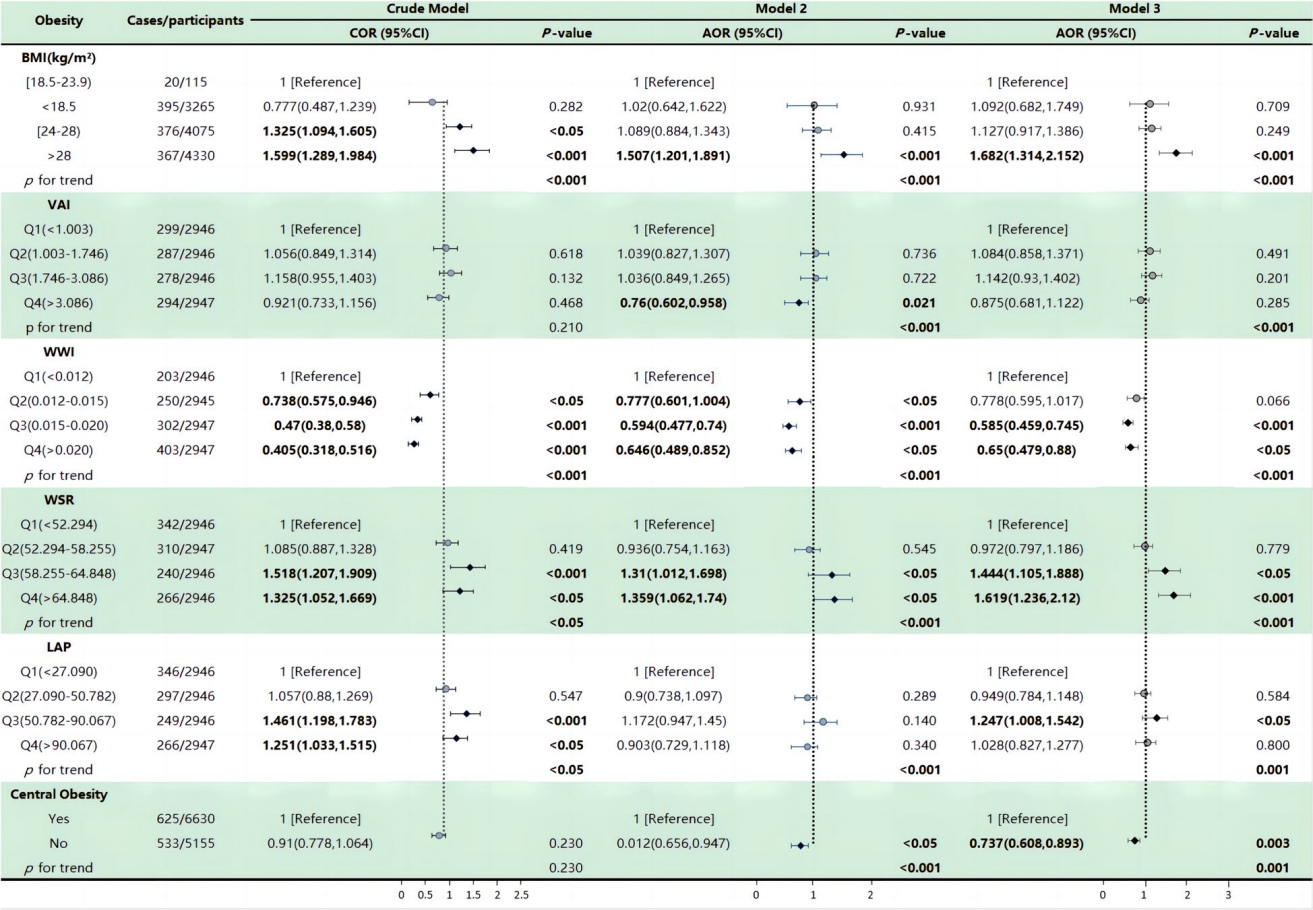
Regression analyses of the association between obesity index and constipation, weighted. Crude Model was not adjusted for any confounding variables. Model 2 was adjusted for gender, age and ethnicity. Model 3 was adjusted for gender, age, ethnicity, education, ratio, diabetes, smoking, alcohol use, hypertension, sleep duration, depression, physical activity and dietary intake of energy, protein, carbohydrate, dietary fiber, total fat, total saturated fatty acids, total monounsaturated fatty acids, total polyunsaturated fatty acids, cholesterol, phosphorus, magnesium, selenium and caffeine. COR (95% CI), and AOR (95% CI) in bold indicate statistical significance

After adjusting for weighted data in the crude model, we observed that three indices were positively associated with the risk of constipation: BMI (COR = 1.60, 95% CI: 1.29, 1.98), WSR (COR = 1.33, 95% CI: 1.05, 1.67), and LAP (COR = 1.25, 95% CI: 1.03, 1.52). Conversely, another index, WWI (COR = 0.41, 95% CI: 0.32, 0.52), exhibited a negative correlation with constipation (Fig. 2).

Upon further adjustment and weighting for age, gender, race/ethnicity, and other relevant covariates, we used the lowest quartile of obesity indices as the reference. It was observed that constipation was significantly positively associated with obesity indices in the third and fourth quartiles, including WSR (AOR = 1.31, 95% CI: 1.01, 1.70) and WSR (AOR = 1.36, 95% CI: 1.06, 1.74). Conversely, constipation was significantly negatively associated with obesity indices in the fourth quartile, specifically VAI (AOR = 0.76, 95% CI: 0.60, 0.96), as well as the second, third, and fourth obesity indices: WWI (AOR = 0.78, 95% CI: 0.60, 1.00), WWI (AOR = 0.59, 95% CI: 0.48, 0.74), and WWI (AOR = 0.65, 95% CI: 0.49, 0.85). Additionally, when using BMI as the reference value, constipation was found to be positively associated with obesity indices in the fourth quartile, represented by BMI (AOR = 1.51, 95% CI: 1.20, 1.89).

In the fully adjusted and weighted model, where we still used the lowest quartile of obesity indices as the reference, we found significant positive associations with obesity indices in the third quartile, such as WSR (AOR = 1.44, 95% CI: 1.11, 1.89) and LAP (AOR = 1.25, 95% CI: 1.01, 1.54), as well as obesity indices in the fourth quartile, particularly WSR (AOR = 1.62, 95% CI: 1.24, 2.12). Furthermore, constipation exhibited a significant negative association with central obesity indices, denoted as central obesity (AOR = 0.74, 95% CI: 0.61, 0.89), and with obesity indices in the third quartile, specifically WWI (AOR = 0.65, 95% CI: 0.48, 0.88), and obesity indices in the fourth quartile, including WWI (AOR = 0.59, 95% CI: 0.46, 0.75) and LAP (AOR = 1.25, 95% CI: 1.01, 1.54). Moreover, when using BMI as the reference, constipation exhibited a significant positive association with obesity indices, represented as BMI (AOR = 1.68, 95% CI: 1.31, 2.15).

In the restricted cubic spline analysis within the fully adjusted model, we identified that BMI, WSR, and LAP had a significant non-linear relationship with constipation. On the other hand, WWI and WC showed a significant linear relationship with constipation, with WWI negatively correlated with constipation, while WC was positively correlated with constipation. Importantly, in this study, neither RCS (Fig. 4) nor logistic regression revealed a significant relationship between VAI and the prevalence of constipation.

### Stratified analysis of obesity and constipation

**Fig. 3:**
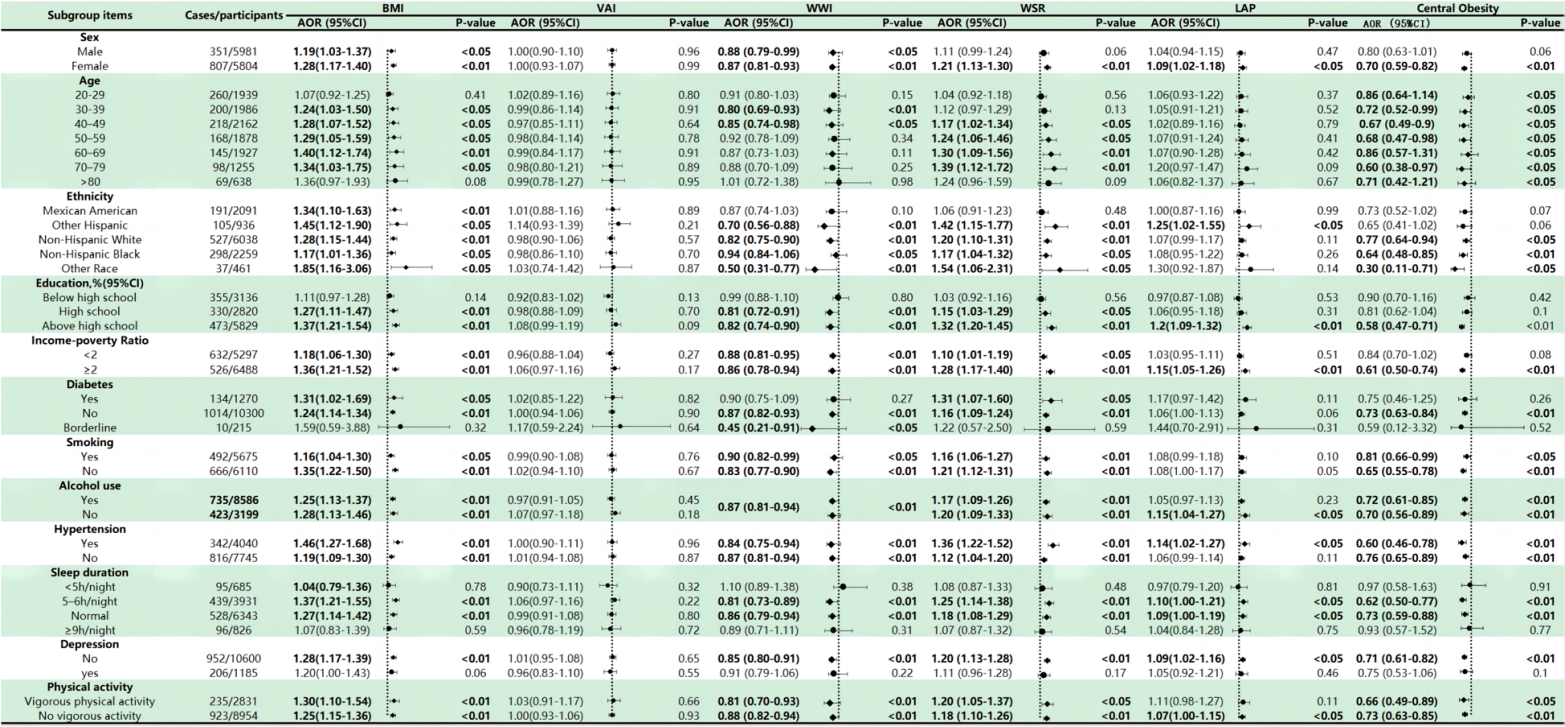
Correlation of generalized weighted quantiles and stratification by health variables with constipation in regression

**Fig. 4:**
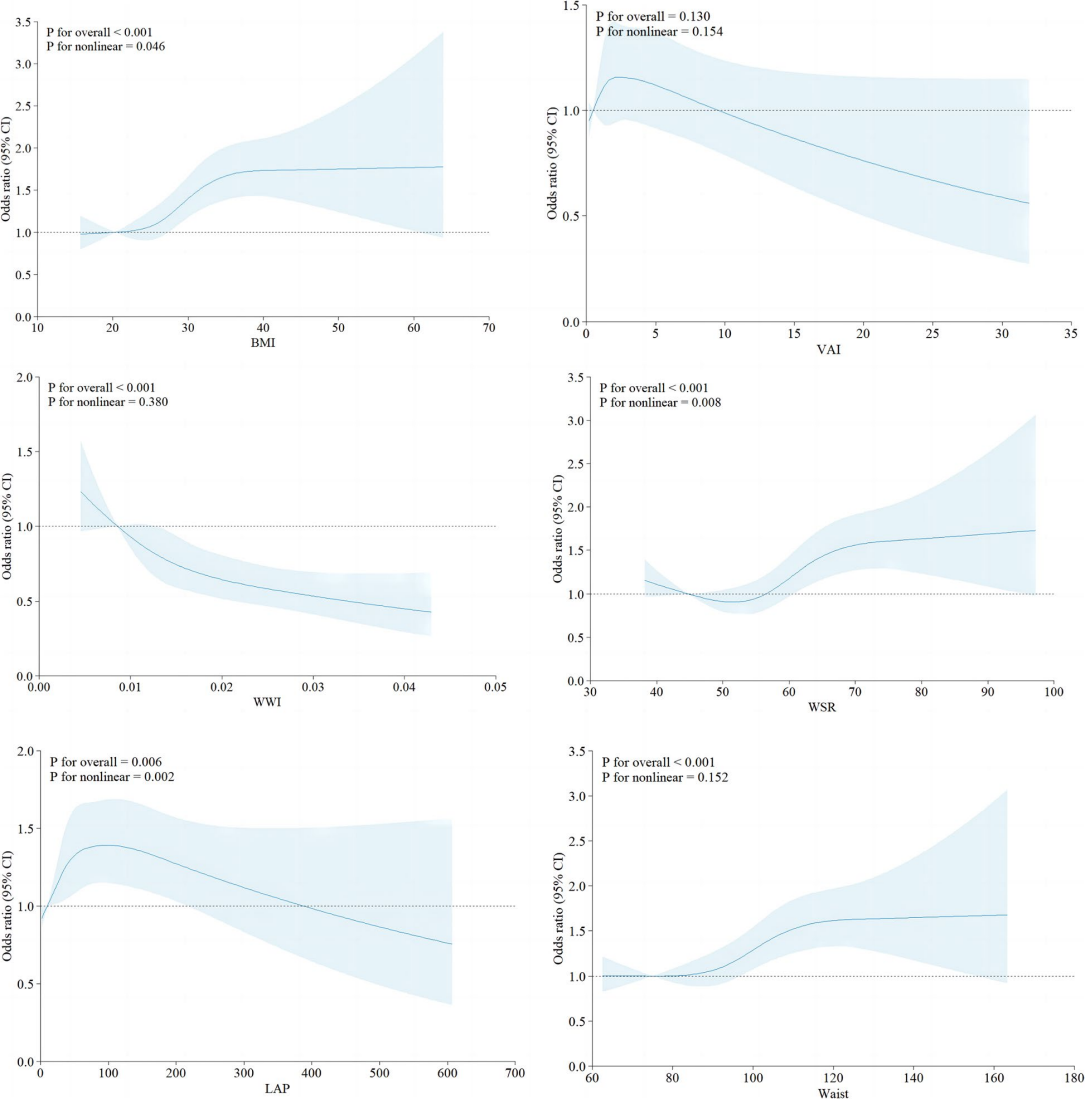
The restricted cubic spline plots of associations of constipation with BMI, VAI, WWI, WSR, LAP and waist circumference

Figure 3 presents a subgroup analysis of six obesity indices and their association with constipation. Stratified analysis reveals dose–response relationships between BMI, VAI, WWI, WSR, LAP, and WC, and the occurrence of constipation (Fig. 4). These associations remained consistent across various subgroups stratified by gender, age, race, education level, poverty ratio, diabetes, smoking, hypertension, sleep duration, depression, and vigorous exercise.

## Discussion

This groundbreaking large-scale study explores the correlations between various obesity indices and constipation in the American population. After adjusting for relevant variables, we identified different trends in various obesity indices and constipation. BMI is widely used in epidemiological studies to assess the risk of health outcomes associated with different weight levels. In this study, RCS analysis identified a significant non-linear relationship between BMI and constipation. As BMI increases, the occurrence of constipation decreases. However, when BMI exceeds 28 kg/m^2^, the risk of constipation significantly increases.

WSR also exhibits a significant non-linear relationship with constipation. When WSR is greater than, 58.3 there is a significant increase in the risk of constipation compared to the first quartile (*p* < 0.05). Interestingly, a previous survey of the Dunedin population in New Zealand found no significant relationship between WSR and constipation [7].

LAP demonstrates an “n”-shaped trend in relation to the risk of constipation, with the highest risk occurring in the third quartile of LAP. Several potential mechanisms may help explain this observed trend. In contrast, a study involving 354 constipated patients showed a significant negative correlation between BMI and colonic transit time [19]. Specifically, constipated patients in the overweight group had shorter rectosigmoid and total colonic transit times compared to patients in the normal BMI group. This relationship may be attributed to bile acid metabolism dynamics. Improvement in colonic transit, stool consistency, and frequency of defecation was observed when functional constipation patients were treated with drugs that affect the levels and transport of bile acids.

A study of 120,000 New Zealand residents found a negative association between overweight and abdominal pain and constipation (OR 0.4, 95% CI 0.2–0.9; *p* = 0.02). The research also revealed a significant negative correlation between WWI > 0.015 and the onset of constipation. When central obesity is absent, the risk of constipation decreases (*P* < 0.05). BMI is the traditional parameter for assessing obesity; however, it cannot differentiate between lean mass and fat mass [20–22]. WC has been proposed as an alternative measure to indirectly assess visceral fat accumulation. Park et al. introduced a new obesity index called weight-adjusted waist index (WWI), which standardizes waist circumference (WC) by body weight and is easy to measure. Therefore, WWI can capture the benefits of WC and attenuate its correlation with BMI, primarily reflecting central obesity independent of body weight.

Many studies have assessed the relationship between constipation and the risk of overweight/obesity; however, the results are controversial. Some studies have proposed opposing conclusions. Pawlowska found no significant differences in weight/BMI between FC patients and control groups [23]. Furthermore, three other studies indicated that individuals with chronic constipation were more likely to be underweight [20–22].

Currently, there is still a lack of large-scale relevant studies on the relationship between VAI, WWI, LAP, WC, and the risk of constipation. Our study also has certain limitations. It is a cross-sectional study; therefore, the absence of cohort studies may lead to a relatively high risk of bias. Further research should focus on investigating the potential mechanisms underlying the association between these two conditions and establish systematic approaches for the treatment of FC and prevention of overweight/obesity to reduce the development of these two conditions. Therefore, carefully planned longitudinal studies are essential to firmly establish the relationship and causality between BMI and constipation.

## Conclusion

In summary, this large-scale pioneering study has examined the relationship between various obesity indicators, such as BMI, VAI, WWI, WSR, LAP, and central obesity, and their association with constipation. These indicators collectively provide a comprehensive assessment of obesity from different bodily perspectives. The study notably found that a reduction in BMI, WSR, LAP and WC is associated with a decreased risk of constipation. In contrast, elevated levels of WWI correlate with a lower constipation risk, a trend that persists even after adjusting for a broad spectrum of variables. These findings not only aid individuals in managing constipation through self-care but also offer healthcare professionals novel insights into the most appropriate treatment of constipation in patients with varying levels of obesity.

## Data Availability

More information about the NHANES could be obtained at: http://www.cdc.gov/nhanes.

## Abbreviations

RCS: Restricted Cubic Spline
BSFS: Bristol Stool Form Scale
VAI: Visceral Adiposity Index
WWI: Weight-adjusted-waist Index
WSR: Waist-stature Ratio
LAP: Lipid Accumulation Products
BMI: Body Mass Index
PIR: Poverty Income Ratio
WC: Waist Circumference
NHANES: National Health and Nutrition Examination Survey
